# Carbide Precipitation during Tempering and Its Effect on the Wear Loss of a High-Carbon 8 Mass% Cr Tool Steel

**DOI:** 10.3390/ma11122491

**Published:** 2018-12-07

**Authors:** Shaoying Li, Xiaojun Xi, Yiwa Luo, Mingtao Mao, Xiao Shi, Jing Guo, Hanjie Guo

**Affiliations:** 1 School of Metallurgical and Ecological Engineering, University of Science and Technology Beijing, Beijing 100083, China; 15081659279@163.com (S.L.); xiyikun123@126.com (X.X.); lyw918@126.com(Y.L.); 18046511210@163.com (M.M.); mighty_ty_works@163.com (X.S.); guojing@ustb.edu.cn (J.G.); 2 Beijing Key Laboratory of Special Melting and Preparation of High-End Metal Materials, Beijing 100083, China

**Keywords:** high-carbon 8 mass% Cr tool steel, different tempering temperatures, wear resistance, carbide precipitation

## Abstract

In this paper, the precipitation of carbide and wear loss of high-carbon 8 mass% Cr tool steel at two tempering conditions (i.e., 773–803 K and 823–853 K) were studied by INCA Steel, EPMA-1720H, XRD, and ML-10 tester. The results show that the particles of test steels include the carbides (Cr_7_C_3_ and Cr_23_C_6_) and carbides nucleated on Al_2_O_3_. When carbides are of the same size, the number of carbides in test steel at a tempering temperature of 773–803 K is greater than that at a tempering temperature of 823–853 K, especially when the size of carbides is less than 5 μm. Compared with the test steel tempered at 823–853 K, the distance between adjacent actual particles reduced by 80.6 μm and the maximum amount of reduction was 9.4% for single wear loss at the tempering temperature of 773–803 K. It can be concluded from thermodynamics results that Al_2_O_3_ inclusions began to precipitate in liquid, and the precipitation of carbides was at the solid–liquid region. Al_2_O_3_ can be used as the nucleation interface of carbide, thus promoting the formation of carbides. During the cooling of molten steel, a lower temperature can increase the difference of actual solubility product bigger than equilibrium solubility product, thus promoting the carbide formation.

## 1. Introduction

In recent years, cutting dies, forming dies, gauges, and other tool steel have attracted attention in the critical applications of cold-worked tool steels [1]. The typical application of this steel is the cutter ring in the TBM (Tunnel Boring Machine), which is used in tunnel excavation in various environments such as cities, seabeds, and mountains. The special use environment requires materials with excellent wear resistance. At present, the methods for improving wear resistance include producing a metal matrix composite (MMC) by spraying carbide on a metal surface, and inlaying a hard alloy outside the steel matrix [2,3]. But these costs are much higher than ordinary steel materials.

For the steel material matrix, studies showed that the distribution of carbides played an important role in improving wear resistance [4,5,6]. In the electroslag remelting–continuous rapid solidification (ESR-CRS) process, increasing water-cooling pressure is beneficial to reducing the formation of primary carbides. The precipitation of primary carbide and grain boundary cementite can be effectively inhibited by controlling the water cooling pressure above 0.06 MPa. [7]. In the austenitizing process, due to the nonequilibrium segregation behavior, temperature affects the total boron concentration of austenite grain boundaries, and the increase in boron concentration enlarges the temperature range of M_23_(C, B)_6_ precipitation [8]. After austenitization, an increase of cooling rate can decrease the volume fraction of carbide; meanwhile, the size distribution of the carbides is meliorated [9]. During subzero treatment, lowering of temperature is beneficial to reducing retained austenite content related to secondary carbide particles. [10].

Surely, the methods of controlling carbide precipitation also include spheroidization [11,12], control of hot-pressing conditions for carbide fracture [13], tempering [14], and so on. In recent years, some scholars have studied the precipitation of carbides in these processes. For example, the precipitation of carbides in medium-manganese steel during tempering was predicted by thermodynamics and kinetics simulations. The results showed that cosegregation of Mn and C is a precondition for the precipitation of carbide and its precipitation is attached to the core [15]. For 316H stainless steel, the precipitation of grain boundary carbides and its size distribution at a temperature range of 673–1073 K during service were studied by the Kampmann and Wagner numerical framework. It was reported that temperature has an effect on the size of carbides [16].

However, systematic studies on the effects of tempering temperature on carbide precipitation and wear resistance have rarely been reported for high-carbon 8 mass% Cr tool steel. In this paper, the tool steel is taken as an example to study the influence of tempering temperature on carbide precipitation control from the perspective of precipitation thermodynamics. The analysis of carbide size, quantity, and morphology was carried out by means of automatic analysis system of inclusions and electron probe. Finally, the wear resistance was tested to determine the effect of carbide precipitation control on wear resistance during tempering.

## 2. Experiment

### 2.1. Material

The selected tool steel grade, high-carbon 8 mass% Cr tool steel, was provided by a steel group (Tianjin Cisri-Harder Materials & Technology Co., Ltd., Tianjin) in China. Table 1 shows the chemical composition of the test steel. Among these elements, alloying elements such as chromium, molybdenum, and vanadium are all strong carbide-forming elements. For the test steel, higher Cr and C percentages promoted the formation of eutectic crude chromium carbides during solidification. Appropriate hardening to 60 HRC can achieve a compression yield strength of up to 2350 MPa [17].

### 2.2. Heat Treatment

The parameters of the heat treatment are shown in Table 2.

The test steel was divided into two groups, one of which was processed according to process A, and the final hardness was 58–60 HRC. The other group was processed according to process B, and the final hardness was 61–63 HRC. The whole heat treatment process included spheroidizing annealing, austenitizing, and two tempering stages. In the austenitizing process, oil bath protection was performed to prevent decarburization of the sample surface. Heating of sample was conducted in a BLMT-1600 °C tubular furnace (Luoyang braveman special testing furnace Co., Ltd., Luoyang, China). In order to ensure low oxygen content in the furnace, purified argon gas was fed from the bottom of the furnace at a constant flow rate of 100 L/h. A double Pt-Rh thermocouple of type B was placed in the furnace to control the temperature within ±1 K.

### 2.3. Microstructural Characterization

The two samples after different tempering were polished and etched with 4% nital solution to observe the microstructures under optical microscope (OM) (Leica, Wetzlar, Germany). The microstructures of the unetched samples were observed under electron-probe microanalysis EPMA-1720H (EPMA) (JEOL, Kyoto, Japan) as well as scanning electron microscope (SEM, Carl-Zeiss, Oberkochen, Germany) in secondary electron mode. The size distribution and number of the precipitation particles were measured by automated inclusion analysis system INCA (INCA Steel) (Oxford Instruments, Oxfordshire, UK). The minimum particle size which INCA Steel could detect was 1 μm on the surface of a specimen of 4 mm × 4 mm. It is worth noticing that the size is the equivalent diameter of each carbide in the measured field. The types of carbides were determined by X-ray diffraction-MXP21VAHF (XRD) (M21X, MAC Science Co., Ltd., Tokyo, Japan).

### 2.4. Tribological Testing and Examination Techniques

**Figure 1:**
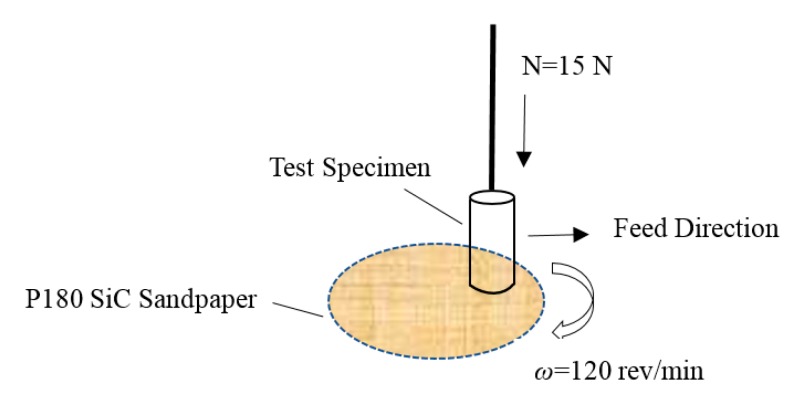
Schematic of wear test specimen and sandpaper.

A testing specimen was sectioned from each of samples A and B to a cylinder of 4 mm diameter and 25 mm length and subsequently achieved ≤3.2 μm average surface roughness (Ra) by plate grinding. The wear tests were conducted on an abrasive wear tester ML-10 (Xuanhua Material Testing Machine Factory, Zhang Jiakou, China) using silicon carbide waterproof abrasive sandpaper. The device schematic is shown in Figure 1. The rotation speed of the sandpaper was 120 rev/min. The feed rate of the cylinder was 2 mm for the circle. The test distance was 19.69 m, with pressure at 1.19 MPa (15 N). It is worth mentioning that five friction tests were carried out for the same cylinder of sample A or B. A new waterproof abrasive sandpaper was used for every test. The wear loss of sample was measured by TG328B analytical balance (Shanghai Yueping Scientific Instrument Co., Ltd., Shanghai, China). For the analytical balance, the range of measurement was 0–200 mg, and the precision was 0.1 mg. 

## 3. Results

### 3.1. Precipitates Formation in High-Carbon 8 Mass% Cr Tool Steel Calculated by Thermo-Calc

In high-carbon 8 mass% Cr tool steel, phase transformation and precipitation of carbides can be calculated by Thermo-Calc with the TCFE7 database.

**Figure 2:**
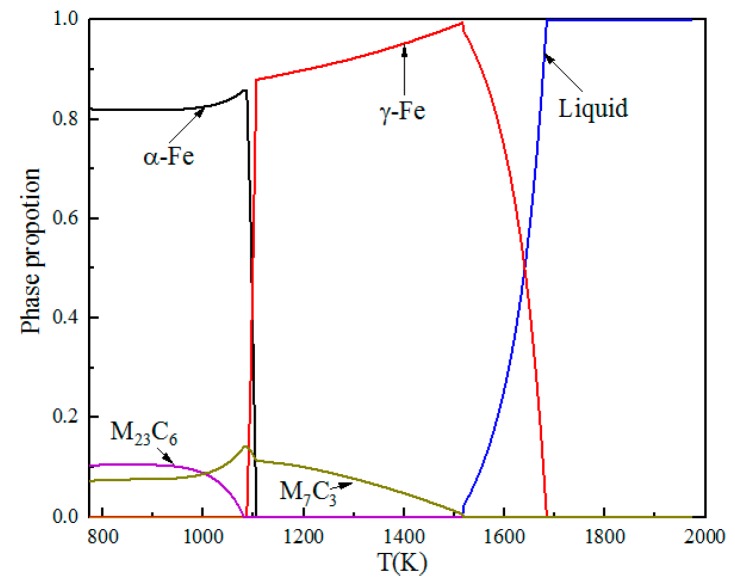
Phase equilibrium diagram of the steel calculated using Thermo-Calc software (M represents metallic element atom; C represents carbon atom).

Figure 2 shows the phase stability diagram during the solidification of liquid steel. When the steel was slowly cooled to 773 K, two kinds of carbides were precipitated from high-carbon 8 mass% Cr tool steel, namely M_7_C_3_ and M_23_C_6_. The formation of M_7_C_3_ was prior to M_23_C_6_. The starting temperatures of M_7_C_3_ and M_23_C_6_ were 1513 K and 1093 K, respectively. As the temperature decreased, the element contents of the carbides changed.

**Figure 3:**
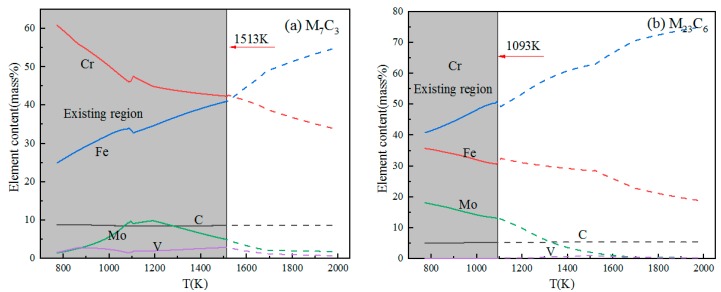
Changes in element contents of M_7_C_3_-type and M_23_C_6_-type carbide in high-carbon 8 mass% Cr tool steel as a function of temperature: (**a**) M_7_C_3_-type carbide; (**b**) M_23_C_6_-type carbide.

The series in Figure 3 show the change in the element contents of carbides as a function of temperature. The elements of Cr, Fe, Mo, and C were contained in both M_7_C_3_ and M_23_C_6_ carbides. However, Cr and Fe are the main components in carbides. M_7_C_3_ began to precipitate at 1513 K. A decrease of the temperature made the Cr content increase but the Fe content decrease. The Mo content increased firstly and then decreased from 1513 K to 773 K. For the carbide of M_23_C_6_, the precipitation temperature was 1093 K. With the decrease of temperature, the Fe content decreased, while the Cr and Mo contents increased. Compared with the Fe content and Cr content, the Mo content and V content of the M_7_C_3_ and M_23_C_6_ carbides were relatively low. M_7_C_3_ and M_23_C_6_ in high-carbon 8 mass% Cr tool steel were found in the previous study [18].

### 3.2. Morphology of Carbides or the Mixture of Carbides and Oxides

#### 3.2.1. Optical Microscope (OM)

**Figure 4:**
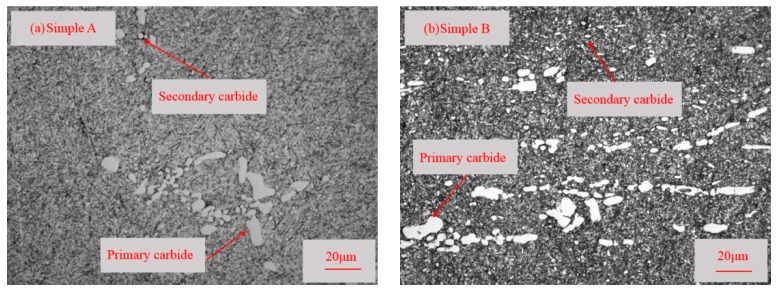
Metallographic observation of the microstructures of specimens: (**a**) sample A; (**b**) sample B.

The microstructures of etched specimens that were tempered at different temperatures were observed by OM as shown in Figure 4.

The observation shows that the microstructure consists of tempered martensite, retained austenite, primary carbides in martensite, and secondary carbides in a matrix of martensite. This phenomenon is the same as the thermodynamic calculation results using Thermao-Calc software in Section 3.1. Based on the carbide’s size, the carbides can be classified as primary carbides (>5 μm) and secondary carbides (<5 μm). The classification of carbides have been detailed in the literature [19,20,21]. In general, small-sized primary carbides are identified as secondary carbides, but the number of such carbides is negligible. In Figure 4, large, white primary carbides were rod-shaped, and tiny, white secondary carbides were spherical. Moreover, it can be seen that compared with sample A, sample B, which tempered at a lower temperature, contains a larger amount of secondary carbides.

#### 3.2.2. Scanning Electron Microscope (SEM)

**Figure 5:**
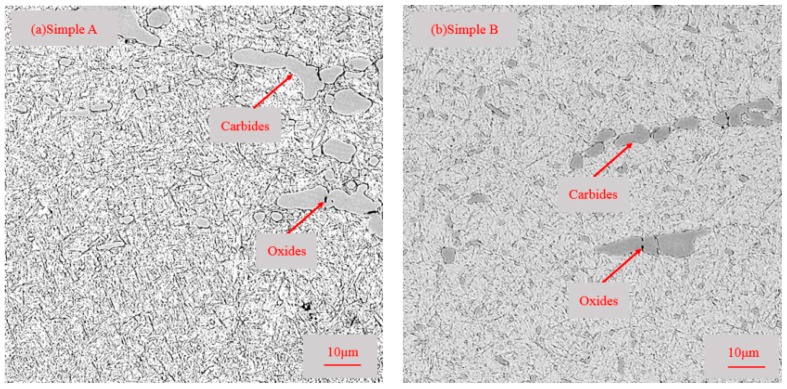
Carbides and oxides SEM morphology: (**a**) sample A, (**b**) sample B.

Some characteristics in the microstructure due to different tempered temperatures were not observed at low magnifications as shown in Figure 4. Very fine particles can be found in Figure 5.

The carbides and oxides observed in samples A and B are shown in Figure 5. There were individual carbides and carbides nucleated on oxides for both sample A and sample B. It can be inferred from Figure 5 that the presence of oxides core promoted the nucleation and growth of carbides. EPMA was carried out to further observe the elements’ distribution in the carbides nucleated on oxides.

#### 3.2.3. Electron-Probe Microanalysis (EPMA)

The EPMA measurements show strong segregation of alloying elements such as aluminum, silicon, oxygen, carbon, chromium, molybdenum, and vanadium at the oxide and carbide.

**Figure 6:**
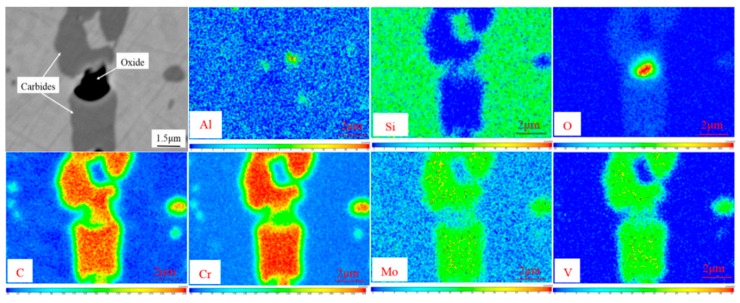
Carbides and oxides elemental maps of sample B.

The elemental segregation has taken place at the boundaries between oxide and carbide. The results of the elemental plots reveal that elemental partitioning of sample A was the same as that of sample B. According to Figure 6, the oxide was Al_2_O_3_/SiO_2_ and the carbides were (Fe, Cr)-rich Cr_7_C_3_ or Cr_23_C_6_ for both samples A and B. The carbides contained small amounts of molybdenum and vanadium, which is consistent with changes in the content of the M_7_C_3_ and M_23_C_6_ elements in Figure 3. Carbide nucleated on oxide could be attributed to the precipitation thermodynamics. This part will be discussed in Section 4.

#### 3.2.4. Size and Distribution

**Figure 7:**
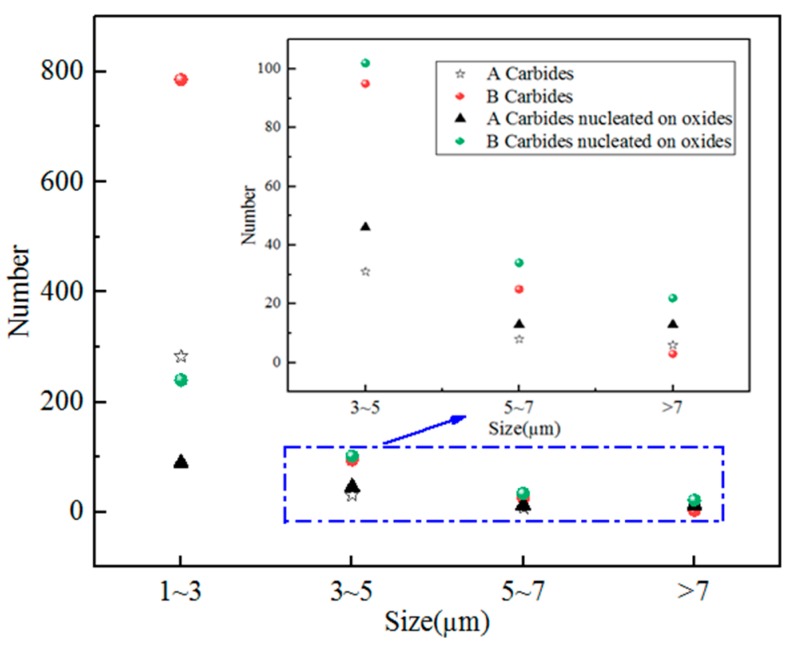
Number distribution of various sizes for carbides and carbides nucleated on oxides.

The details that include the size distribution and number of the carbides and carbides nucleated on oxides measured by INCA Steel are given in Figure 7.

In Figure 7, the abscissa represents the size of precipitations and the ordinate represents its number. It can be seen that under the same size, the number of carbides (or carbides nucleated on oxides) at tempering temperature 773–803 K was more than that at the tempering temperature 823–853 K, especially when the size was less than 5 μm. Therefore, it could be inferred that low tempering temperature was beneficial to enlarge the number of the carbides. Moreover, an increase of size decreases the number of precipitations. Most of the precipitates were less than 5 μm, which are considered to be secondary carbides, and few carbides were more than 7 μm. This indicates that most carbides precipitate during tempering. This phenomenon could be attributed to the holding time and temperature. In general, coarse carbides precipitated from liquid steel or solid–liquid steel, but when the time of nucleation and growth was not enough, coarse carbides could not form in liquid steel or solid–liquid steel.

#### 3.2.5. Type of Carbides

**Figure 8:**
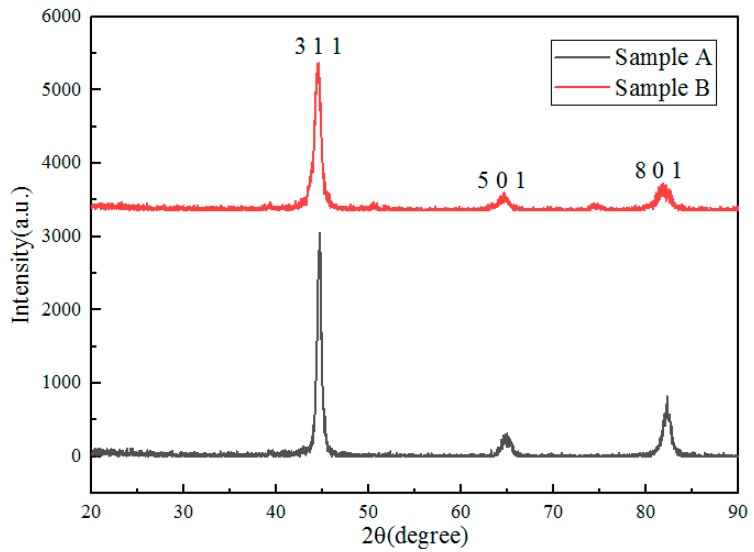
Experimental XRD profiles (relative intensities) of samples A and B.

In order to determine the types of carbides, XRD was conducted. The result showed that the type of carbides was M_7_C_3_. M_23_C_6_ was not found from Figure 8. The reason is that the carbides’ type transformed from M_7_C_3_ to M_23_C_6_. It can be seen from Figure 2 that when M_23_C_6_ begins to form, the content of M_7_C_3_ decreases gradually. The precipitation temperature of M_23_C_6_ is lower than that of M_7_C_3_. From the kinetic point of view, the low temperature is not conducive to the growth of M_23_C_6_. The reason for this is that the rate of reaction is exponential with temperature on the basis of the Arrhenius formula [22]. So, in test steel, M_7_C_3_ shows the stronger intensity and clarity of crystalline diffraction lines than M_23_C_6_ in XRD. However, M_23_C_6_ was found in EPMA.

**Figure 9:**
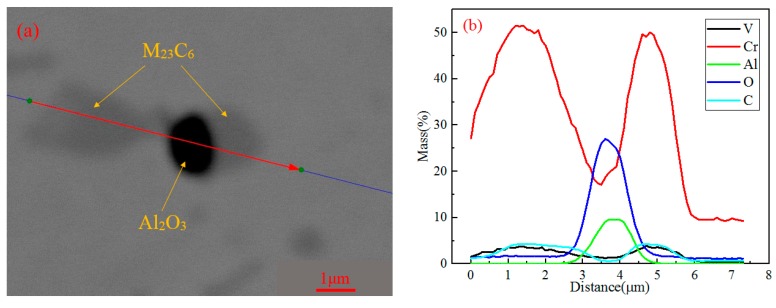
EPMA profiles obtained across carbides and oxides of sample B: (**a**) measurement path; (**b**) V, Cr, Al, O, and C.

In order to obtain the content of elements in carbides, the carbides nucleated on oxides were analyzed by line scanning. Figure 9a shows the morphology of carbides nucleated on oxide. From Figure 9b, it can be seen that the black core is Al_2_O_3_, and the gray particles around it are chromium-rich carbides, in which the chromium content is about 50%, and the carbon content is about 5%. According to the content of carbides calculated using Thermo-Calc in Figure 3b, it can be inferred that the carbides are Cr_23_C_6_. According to phase equilibrium diagram (Figure 2), M_23_C_6_ and M_7_C_3_ are two types of carbides in the steel. Given the above, there are M_7_C_3_ and M_23_C_6_ in the steel.

### 3.3. Wear Resistance

Table 3 presents the mass loss on abrasive wear tests. Since the same cylindrical specimen of sample A or B was subjected to five friction tests, the wear amount of each wear test is defined as a single wear loss, and the mass loss of a cylindrical specimen before and after wear is defined as the total mass loss. As shown in Table 3, for five single wear tests, the single mass loss of sample A was greater than that of sample B, and difference ranged from 0 to 3.5 mg. The total mass loss of A was 6.8 mg more than B, which shows that the wear resistance of sample B is better than that of sample A. Combining the number of carbides and carbides nucleated on oxides, it can be found that an increase in the number of carbides is beneficial to improve wear resistance [23]. The mechanism of this part will be discussed in Section 4.4.

## 4. Discussion

From Section 3, it can be concluded that precipitate types after tempering are mainly Cr_7_C_3_, Cr_23_C_6_. Experimental results in Figure 6 show the morphology of carbides nucleated on oxides. By thermodynamics, the initial generation temperatures of carbides and oxide inclusions are calculated to explain this phenomenon. The solidification of molten steel can be divided into three stages, namely, liquid phase, solid–liquid dual phase, and solid phase. The liquidus temperature (T*_l_*) and solidus temperature (T*_s_*) of test steel are calculated to be 1365 K and 1682 K by the Empirical formula [24], respectively. The results are similar to the calculation of the Thermo-Calc software.

T*_l_* = 1809 − 83*w*(C) − 7.8 *w*(Si) − 5 *w*(Mn) − 32 *w*(P) − 31.5 *w*(S) − 1.5 *w*(Cr) − 2 *w*(Mo) − 2 *w*(V) − 3.6 *w*(Al) − 18*w*(Ti) (1)

T*_s_* = 1809 − 344*w*(C) − 12.3*w*(Si) − 6.8*w*(Mn) − 124.5*w*(P) − 183.5 *w*(S) − 1.4 *w*(Cr) − 4.1*w*(Al) − 4.3*w*(Ni) (2)

where *w* is the percentage of mass concentration for the element.

### 4.1. Formation of Oxides and Carbides in the Liquid Phase

Depending on the composition of molten steel, the possible forms of oxides in the steel include Cr_2_O_3_, Al_2_O_3_, MnO, SiO_2_, V_2_O_3_, and MoO_2_. The Gibbs free energy of formation of oxides in the liquid steel is obtained through the following chemical reaction, as shown in Table 4.

In order to make the reaction (1~6) spontaneously proceed to the right, the condition to be reached is ∆G < 0.

(3) $$\Delta G=\Delta G^{\theta }+\mathrm{RT}\ln\frac{a_{M_{x}O}}{a_{\left[M\right]}^{x}a_{\left[O\right]}}=\Delta G^{\theta }+\mathrm{RT}\ln\frac{a_{M_{x}O}}{f_{\left[M\right]}^{x}{\left[\%M\right]}^{x}f_{\left[O\right]}[\%O]}$$

where $a_{M_{x}O}$, $a_{\left[M\right]}$, $a_{\left[O\right]}$ are the activities of oxides M*_x_*O, M, and O, respectively; [%M] and [%O] are the mass fractions of metal M and oxygen in molten steel, respectively; *f*_[*M*]_ and *f*_[*O*]_ are the activity coefficients of the metal elements M and oxygen, respectively, calculated by the Wagner model.

According to Table 1, except for the high content of Cr, the content of other alloying elements is relatively low. So the mass fraction of the alloying elements is regarded as tending to zero, and the solvent is iron liquid whose concentration is close to 1. The Wagner model can be characterized by a single interaction coefficient, in which the activity coefficient expression of each element is

(4) $$\mathrm{lg}f_{k}=\sum_{j=2}^{n}e_{k}^{j}[\%j]$$

where $e_{k}^{j}$ is the first interaction coefficient between the solute elements, *k* is any solute component of the multiphase, and *j* is the 2, 3, ..., n solute component. The first interaction coefficient of each element in the molten steel for O, Cr, Al, Mn, Si, V, Mo is shown in Table 5 [25].

According to Equation (4) and Table 5, the activity coefficient of each element was obtained, which are *f*_C_ = 1.02, *f*_O_ = 0.06, *f*_Cr_ = 0.71, *f*_Al_ = 1.45, *f*_Mn_ = 0.82, *f*_Si_ = 2.14, *f*_V_ = 0.35, and *f*_Mo_ = 0.76.

**Figure 10:**
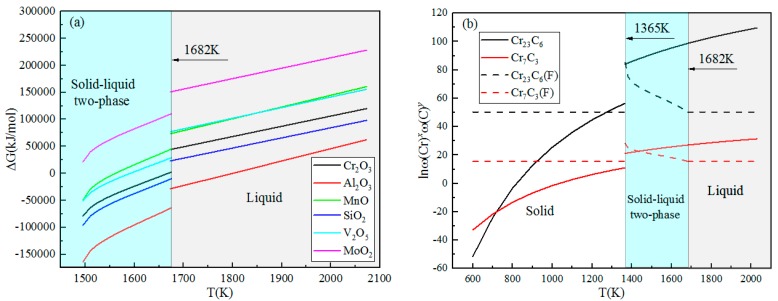
Precipitation thermodynamic calculation results: (**a**) oxides, (**b**) carbides.

In the liquid phase (T > 1682 K), each activity coefficient and concentration are substituted into Equation (3), and the Gibbs free energy of the oxide is generated. The relationship diagram of ∆G ~ T is shown in the liquid zone of Figure 10a. It can be seen that only Al_2_O_3_ is formed in the liquid phase, and other oxides cannot be formed in the liquid phase.

When the actual solubility product of the precipitate-forming element is larger than the equilibrium solubility product, carbide can be formed. The solubility product of Cr_7_C_3_ or Cr_23_C_6_ in liquid steel is deduced as follows:

23[Cr] + 6[C] = Cr_23_C_6_(s), ∆G^θ^ = −887890 + 1284.48T (5)

7[Cr] + 3[C] = Cr_7_C_3_(s), ∆G^θ^ = −356120 + 417.6T (6)

According to the principle of precipitation thermodynamics, the equilibrium solubility product of M*_x_*C*_y_* in liquid steel can be expressed as

(7) $$\ln\left({\left[\%M\right]}^{x}{\left[\%C\right]}^{y}\right)=B+\frac{A}{T}-x\ln f_{M}-y\ln f_{C}$$

According to Equations (5)–(7), the solubility products of Cr_23_C_6_ and Cr_7_C_3_ in liquid steel are obtained as follows:

(8) $$\ln\left({\left[\%\mathrm{Cr}\right]}^{23}{\left[\%C\right]}^{6}\right)=-1284.48+\frac{887890}{T}-23\ln f_{Cr}-6\ln f_{C}$$

(9) $$\ln\left({\left[\%\mathrm{Cr}\right]}^{7}{\left[\%C\right]}^{3}\right)=-417.6+\frac{356120}{T}-7\ln f_{Cr}-3\ln f_{C}$$

Put *f_C_* = 1.02, *f_Cr_* = 0.71 into Equations (8) and (9), and the relationship between ln([%Cr]*^x^*[%C]*^y^*) and T is shown in the liquid zone of Figure 8b. It can be seen that Cr_23_C_6_ and Cr_7_C_3_ cannot be formed in liquid steel.

### 4.2. Formation of Oxides and Carbides in Solid–Liquid Dual Phase

Due to the element segregation in the solidification front, the Scheil formula is introduced in the solidification process as follows:

(10) $$[\%N]=\frac{\left[\%N_{0}\right]}{P_{s}{(K}_{N}-1)+1}$$

(11) $$[\%M]=[\%M_{0}]{(1-P_{s})}^{\left(K_{M}-1\right)}$$

(12) $$P_{s}=\frac{{(T}_{m}-T_{s}{)(T}_{l}-T)}{{(T}_{l}-T_{s}{)(T}_{m}-T)}$$

where [%M] and [%N] are the mass fraction of the metal element M and the nonmetal element N in the solid–liquid phase during solidification;

[%M_0_] and [%N_0_] are the mass fraction of the metal element M and the nonmetal element N in the liquid steel before solidification;

K_M_ and K_N_ are the equilibrium solute partition coefficients for M and N, respectively. In the literature [27], the equilibrium partition coefficients of solute elements oxygen, carbon, vanadium, chromium, molybdenum, and aluminum in the solidification process are 0.02, 0.17, 0.90, 1, 0.80, and 0.60, respectively;

T is the temperature of the system during solidification, K;

T*_m_* is the melting point of pure iron, 1809 K;

T*_l_*, T*_s_* are the liquidus and solidus temperatures of steel, 1682 K and 1365 K, respectively.

Put the oxygen concentration and the metal element concentration calculated by the Scheil equation into Equation (2), and the relationship of oxides between ∆G and T can be obtained, as shown at the solid–liquid two-phase zone of Figure 10a. In the same way, the relationship of carbides between ln([%Cr]*^x^*[%C]*^y^*) and T is shown in Figure 10b. It can be seen from Figure 10a that when the temperature is in the solid–liquid two-phase region, the Gibbs free energy of Al_2_O_3_ is much smaller than other oxides, so Al_2_O_3_ is easily formed. Due to the segregation of solidification, the concentration of the liquid phase in the solid–liquid two phases is greater than the solid phase.

The equilibrium solubility product curves of Cr_7_C_3_ and Cr_23_C_6_ in the liquid phase of the solidification front have intersections with the actual solubility product curves. With the decrease of temperature, the actual solubility product becomes greater than the equilibrium solubility product, and the precipitation thermodynamic condition is reached. The intersection points are the initial precipitation temperatures of precipitates in the solid–liquid two-phase zone, and the theoretical precipitation temperature of Cr_7_C_3_ and Cr_23_C_6_ are 1425 K and 1375 K, respectively, which can be judged by the intersection point [28].

### 4.3. Formation of Carbides in Solid Phase

Since the Gibbs free energy of element dissolution in solid-phase ferrite has not been studied, empirical data in austenite is still used in the calculation process. During the solidification process, high-carbon 8 mass% Cr tool directly enters the austenite zone and has no solute in the high-temperature ferrite zone according to Figure 2. The chemical reaction of Cr forming carbides in austenite can be obtained by Equations (13) and (14).

[Cr]*_L_* = [Cr]_δ_ △G^θ^ ≈ 1046 J/mol (13)

[Cr]*_γ_* = [Cr]_δ_ △G^θ^ ≈ 418 J/mol (14)

where δ represents high-temperature ferrite and γ represents austenite.

According to the equilibrium solid solubility formula of graphite C in austenite,

(15) $$\mathrm{lg}{\left[C\right]}_{\gamma }=3.605-\frac{5386}{T}$$

The free energy of dissolving graphite C into austenite is

C(s) = [C]*_γ_*, △G = 32167.2 − 28.72T (16)

According to Equations (13)–(16), the solubility products of Cr_23_C_6_ and Cr_7_C_3_ in austenite are obtained as follows:

23[Cr]*_γ_* + 6[C]*_γ_* = Cr_23_C_6_(s), △G^θ^ = −959797.2 + 1172.7T (17)

7[Cr]*_γ_* + 3[C]*_γ_* = Cr_7_C_3_(s), △G^θ^ = −389247.6 + 376.98T (18)

Combined with the solubility product formula, the relationship between the solubility product and the temperature can be obtained for Cr_23_C_6_ and Cr_7_C_3_, as shown in Figure 10b. It can be seen that Cr_23_C_6_ and Cr_7_C_3_ begin to precipitate. The temperature is lower and the difference between the actual solubility product and the equilibrium solubility product is larger. The carbide is easier to be formed. This is consistent with the statistical results in Section 3.1, that is, the amount of carbides and carbides nucleated oxides in B is greater than in A. The thermodynamic calculation results of the precipitation of oxides and carbides are in agreement with the observations in Section 3.2. Because Al_2_O_3_ precipitates in the liquid phase, Cr_7_C_3_ and Cr_23_C_6_ begin to precipitate in the solid–liquid two-phase region. Therefore, the phase nucleation of Al_2_O_3_ is precipitated first, and Cr_7_C_3_ and Cr_23_C_6_ are attached to the edge as the core.

### 4.4. The Improvement of Wear Resistance

Al_2_O_3_ inclusion could be taken as heterogeneous nuclei for carbide formation [29,30,31]. Since the surface on the Al_2_O_3_ inclusion is a carbide, it is assumed that in hardness, the carbide nucleated on oxide is the same as the carbide. In the following discussion, both carbides and carbides nucleated on oxides are referred to as particles. S. Wei et al. reported carbides’ dispersed distribution can resist microcutting of hard abrasive particles [23].

**Figure 11:**
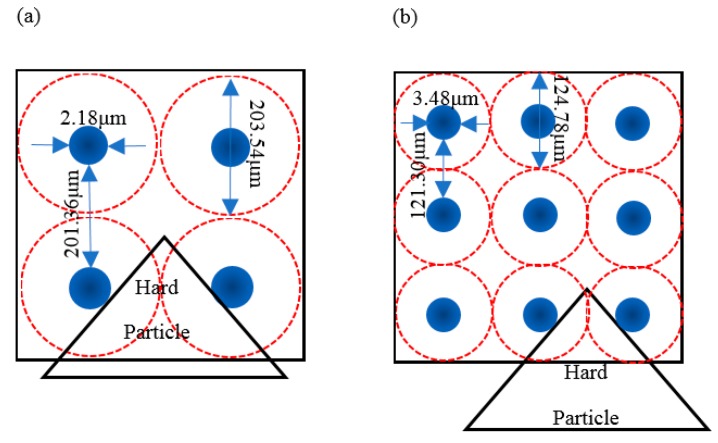
Schematic diagram of carbides in steel and hard particles during wear process: (**a**) sample A, (**b**) sample B.

The distance between carbides decreases and it is more difficult for abrasives to cut into the matrix, so the wear resistance is better [2]. To clearly compare the distance between adjacent particles, related parameters are listed in Table 6. The calculation method is shown in Equations (19) and (20).

(19) $$S_{i}={\sum_{j=1}^{N_{i}}\pi \left(\frac{d_{j}}{2}\right)}^{2}$$

where S*_i_* is the total area occupied by the particles, *i* = 1 represents carbides, and *i* = 2 represents carbides nucleated on oxides. N*_i_* and d*_j_* can be obtained by INCA Steel. Assume that the particles are homogeneously distributed in the test steel with equal diameter D_i_, as shown by the solid circle in Figure 11. The distance Z between the actual adjacent particles is calculated as follows:

(20) $$Z=2\left[\sqrt{\frac{S\times 10^{6}}{\pi \left(N_{1}+N_{2}\right)}}-\sqrt{\frac{S_{1}+S_{2}}{\pi (N_{1}+N_{2})}}\right]$$

where S (mm^2^) is the area of the measured region. According to Section 2.3, the measured area is 4 × 4 mm.

Figure 11 is a schematic diagram of carbides in steel and hard particles during the wear process. According to Equations (1) and (2), the distance Z between the actual adjacent particles was obtained for samples A and B, as shown in Table 6 and Figure 11.

The solid circle represents the actual particle (i.e., carbide of the carbide nucleated oxide) and the dotted circle is the auxiliary line to acquire Z in Figure 11. As can be seen from Figure 11, the distance Z between the actual adjacent particles is 201.36 μm for sample A, and Z for sample B is 121.30 μm. The triangle represents hard particle (i.e., silicon carbide particle). The length of hard particle embedded in the matrix for B is shorter than for A. So the resistance of sample B is prior to sample A. The conclusion is consistent with Zhang’s opinion [2].

## 5. Conclusions

(1) When the size of carbides is the same, the number of carbides in test steel at a tempering temperature of 773–803 K is greater than that at a tempering temperature of 823–853 K, especially when the size of carbides is less than 5 μm.
(2) There are carbides and carbides nucleated on oxides of Al_2_O_3_ in high-carbon 8 mass% Cr tool steel. Al_2_O_3_ precipitates in the liquid phase, Cr_7_C_3_ and Cr_23_C_6_ begin to precipitate in the solid–liquid two-phase region.
(3) For the carbides in the test steel, a decrease in the temperature and an increase in the difference between the actual solubility product and the equilibrium solubility product promote the carbide formation.
(4) The distance between adjacent actual particles is 121.30 μm at the tempering temperature of 773–803 K, which is 80.6 μm shorter than the tempering temperature 823–853 K.
(5) Compared with the test steel tempered at 823–853 K, the maximum amount of reduction is 9.4% for single wear loss at the tempering temperature of 773–803 K.

## Figures and Tables

**Table 1:** Chemical compositions of the test steels (wt %).

Element	Fe	C	Si	Mn	Cr	Mo	V
**Composition** (wt %)	bal.	1.2	1.0	<0.6	8.5	2.50	0.50

**Table 2:** Parameters for the heat treatment process.

Heat Treatment Stage	Temperature (K)/Time (min)	
	Sample A	Sample B
Spheroidizing Annealing	1073–1173/40–60	1073–1173/40–60
Austenitizing	1273–1373/20–40	1273–1373/20~40
First Tempering	823–853/60–90	773–803/60~90
Second Tempering	823–853/60–90	773–803/60–90
Final hardness (HRC)	58–60	61–63

**Table 3:** Mass loss for samples A and B.

Sample	Mass Loss/mg					
	1	2	3	4	5	Total
A	38.3	36.1	37.7	40.4	40.6	193.1
B	37.4	35.8	37.7	38.3	37.1	186.3
*w*(A) − *w*(B)	0.9	0.3	0	2.1	3.5	6.8
[*w*(A) − *w*(B)]/*w*(B)	2.41%	0.84%	0	5.48%	9.4%	3.66%

**Table 4:** Chemical reactions and Gibbs Energy ∆G^θ^.

Number	Reaction	∆G^θ^ (J/mol)
1	2/3[Cr] + [O] = 1/3 Cr_2_O_3_	−273010 + 119.69T [25]
2	2/3[Al] + [O] = 1/3 Al_2_O_3_	−408333.333 + 131.26T [25]
3	[Mn]+[O]= MnO	−244316 + 106.84T [25]
4	1/2[Si] + [O]= 1/2 SiO_2_	−297142.5 + 114.88T [25]
5	2/3[V] + [O]= 1/3 V_2_O_3_	−251164 + 102.24T [26]
6	1/2[Mo] + [O]= 1/2 MoO_2_	−172254.805 + 115.08T [27]

**Table 5:** The first interaction coefficient of each element to C, O, Cr, Al, Mn, Si, V, Mo.

$e_{k}^{j}$	Al	C	Cr	Mn	Mo	N	O	S	Si	V
**C**	0.043	0.14	−0.024	−0.012	−0.0083	0.11	−0.34	0.46	0.08	−0.077
**Cr**		−0.12	−0.0003		0.0018	−0.19	−0.14	−0.02	−0.0043	
**Mn**		−0.07				−0.091	−0.083	−0.048		
**Mo**		−0.097	−0.0003	0.0046		−0.1	−0.0007	−0.0005		
**O**	−3.9	−0.45	−0.04	−0.021	0.0035	0.057	−0.2	−0.133	−0.131	−0.3
**Si**	0.058	0.18	−0.0003	0.002			−0.23	0.056	0.11	0.025
**V**		−0.34				−0.35	−0.97	−0.028	0.042	0.015

**Table 6:** Number, total area of particles, and the distance between adjacent actual particles for samples A and B.

Sample	A	B
**Number of Carbides N_1_**	329	910
**Number of Carbides Nucleated on Oxides N_2_**	163	399
**Total Area of Carbides S_1_ (μm^2^)**	682.04	2498.33
**Total Area of Carbides Nucleated on Oxides S_2_ (μm^2^)**	1150.70	9993.32
**The distance between Adjacent Actual Particles Z (μm)**	201.36	121.30

## References

[1] Masb A.R., Mb M., Nisb H., Msb S. (2018). A comprehensive review on cold work of AISI D2 tool steel. Metall. Res. Technol..

[2] Zhang G.S., Xing J.D., Gao Y.M. (2006). Impact wear resistance of WC/Hadfield steel composite and its interfacial characteristics. Wear.

[3] Nurminen J., Jonne N., Petri V. (2009). Microstructure and properties of hard and wear resistant MMC coatings deposited by laser cladding. Int. J. Refact. Met. Hard Mater..

[4] Bourithis L., Papadimitriou G.D., Sideris J. (2006). Comparison of wear properties of tool steels AISI D2 and O1 with the same hardness. Tribol. Int..

[5] Hetzner D.W., Van Geertruyden W. (2008). Crystallography and metallography of carbides in high alloy steels. Mater. Charact..

[6] Nanesa H.G., Boulgakoff J., Jahazi M. (2016). Influence of prior cold deformation on microstructure evolution of AISI D2 tool steel after hardening heat treatment. J. Manuf. Process.

[7] Du G., Li J., Wang Z.B. (2017). Control of carbide precipitation during electroslag remelting-continuous rapid solidification of GCr15 Steel. Metall. Mater. Trans. B.

[8] Hwang B., Suh D.W., Kim S.J. (2011). Austenitizing temperature and hardenability of low-carbon boron steels. Scr. Mater..

[9] Luo Y.W., Guo H.J., Guo J. (2018). Effect of cooling rate on the transformation characteristics and precipitation behaviour of carbides in AISI M42 high-speed steel. Ironmak. Steelmak..

[10] Das D., Ray K.K., Dutta A.K. (2009). Influence of temperature of sub-zero treatments on the wear behavior of die steel. Wear.

[11] Zhou X.F., Fang F., Jiang J.Q., Zhu W.L., Xu H.X. (2014). Refining carbide dimensions in AISI M2 high speed steel by increasing solidification rates and spheroidising heat treatment. Mater. Sci. Technol..

[12] Ghomashchi M.R., Sellars C. (1993). Microstructural changes in as-cast M2. Metall. Mater. Trans. A.

[13] Bombac D., Fazarinc M., Podder A.S., Kugler G. (2013). Study of carbide evolution during thermo-mechanical processing of AISI D2 tool steel. J. Mater. Eng. Perform..

[14] Kang H.J., Yoo J.S., Ji T.P., Ahn S.T., Kang N., Cho K.M. (2012). Effect of nano-carbide formation on hydrogen-delayed fracture for quenching and tempering steels during high-frequency induction heat treatment. Mat. Sci. Eng. A Struct..

[15] SLEIPNER Cold Work Tool Steel. https://docplayer.net/49143786-Sleipner-cold-work-tool-steel.html.

[16] Silva A.K., Inden G., Kumar A., Ponge D., Gault B., Raabe D. (2018). Competition between formation of carbides and reversed austenite during tempering of a medium-manganese steel studied by thermodynamic-kinetic simulations and atom probe tomography. Acta Mater..

[17] Xiong Q., Robson J.D., Chang L.T., Fellowes J.W., Smith M.C. (2018). Numerical simulation of grain boundary carbides evolution in 316H stainless steel. J. Nucl. Mater..

[18] Kim H., Kang J.Y., Son D., Lee T.H., Cho K.M. (2015). Evolution of carbides in cold-work tool steels. Mater. Charact..

[19] Ko D.C., Kim S.G., Kim B.M. (2015). Influence of microstructure on galling resistance of cold-work tool steels with different chemical compositions when sliding against ultra-high-strength steel sheets under dry condition. Wear.

[20] Das D., Dutta A.K., Ray K.K. (2009). Influence of varied cryotreatment on the wear behavior of AISI D2 steel. Wear.

[21] Fukaura K., Yokoyama Y., Yokoi D., Tsujii N., Ono K. (2004). Fatigue of cold-work tool steels: effect of heat treatment and carbide morphology on fatigue crack formation, life, and fracture surface observations. Metall. Mater. Trans. A.

[22] Keith J.L. (1984). The Development of the Arrhenius Equation. J. Chem. Educ..

[23] Wei S.Z., Zhu J.H., Xu L.J. (2005). Research on wear resistance of high speed steel with high vanadium content. Mat. Sci. Eng. A Struct..

[24] Wang J.Q., Huang J.R. (1977). Solidification of Metal and Its Control.

[25] Huang X.G. (2011). Principle of Steel Metallurgy.

[26] Chen J.X. (1990). Steel Metallurgy (Steelmaking Part).

[27] Chen J.X. (2006). Steelmaking Common Chart Data Manual.

[28] Ning A.G. (2015). Investigation on Nanoscale Precipitates in Hot-Work Die Steel and Comprehensive Strengthening Mechanism of Steel.

[29] Elfawkhry M.K., Fathyk A.M., Eissa M.M. (2014). Effect of Ca-Si modifiers on the carbide precipitation of as-cast hadfield steel. Steel Res. Int..

[30] Li J., Li J., Shi C.B., Wang L.L., Wu Z., Wang H. (2016). Effect of trace magnesium on carbide improvement in H13 steel. Can. Metall. Q..

[31] Quested P.N., Mclean M. (1984). Solidification morphologies in directionally solidified superalloys. Mater. Sci. Eng..

